# Potential of Chitosan/Gelatin-Based Nanofibers in Delivering Drugs for the Management of Varied Complications: A Review

**DOI:** 10.3390/polym17040435

**Published:** 2025-02-07

**Authors:** Popat Mohite, Abhijeet Puri, Shubham Munde, Roshan Dave, Showkhiya Khan, Riteshkumar Patil, Anil Kumar Singh, Pratchaya Tipduangta, Sudarshan Singh, Chuda Chittasupho

**Affiliations:** 1 AETs St. John Institute of Pharmacy and Research, Palghar 401404, Maharashtra, India; mohitepb@gmail.com (P.M.); abhijeetp@sjipr.edu.in (A.P.); shubhamvmunde@gmail.com (S.M.); 122roshan4001@sjipr.edu.in (R.D.); showkhiya@outlook.com (S.K.); riteshkumar280201@gmail.com (R.P.); 2 United Institute of Pharmacy, Prayagraj 211010, Uttar Pradesh, India; singhanil2682@gmail.com; 3 Department of Pharmaceutical Sciences, Faculty of Pharmacy, Chiang Mai University, Chiang Mai 50200, Thailand; pratchaya.t@cmu.ac.th; 4 Office of Research Administration, Chiang Mai University, Chiang Mai 50200, Thailand

**Keywords:** biopolymers, gelatin, chitosan, electrospinning, nanofibers, nanotechnology

## Abstract

Drug delivery systems have revolutionized traditional drug administration methods by addressing various challenges, such as enhancing drug solubility, prolonging effectiveness, minimizing adverse effects, and preserving potency. Nanotechnology-based drug delivery systems, particularly nanoparticles (NPs) and nanofibers (NFs), have emerged as promising solutions for biomedicine delivery. NFs, with their ability to mimic the porous and fibrous structures of biological tissues, have garnered significant interest in drug-delivering applications. Biopolymers such as gelatin (Ge) and chitosan (CH) have gained much more attention due to their biocompatibility, biodegradability, and versatility in biomedical applications. CH exhibits exceptional biocompatibility, anti-bacterial activity, and wound healing capabilities, whereas Ge provides good biocompatibility and cell adhesion properties. Ge/CH-based NFs stimulate cellular connections and facilitate tissue regeneration owing to their structural resemblance to the extracellular matrix. This review explores the additive methods of preparation, including electrospinning, force pinning, and template synthesis, focusing on electrospinning and the factors influencing the fiber structure. The properties of Ge and CH, their role in drug release, formulation strategies, and characterization techniques for electrospun fibers are discussed. Furthermore, this review addresses applications in delivering active moieties in the management of orthopedics and wound healing with regulatory considerations, along with challenges related to them. Thus, the review aims to provide a comprehensive overview of the potential of Ge/CH-based NFs for drug delivery and biomedical applications.

## 1. Introduction

A drug delivery system (DDS) is a formulation or device that facilitates the administration or release of a drug substance into the body, enhancing its effectiveness and safety by regulating the timing, location, and rate of drug release. This involves administering the therapeutic product, releasing its active ingredients, and transporting them across biological membranes to the target site [1]. Novel DDSs have transformed traditional drug administration methods by addressing a range of challenges. These systems enhance the solubility of drugs, prolong their effectiveness, minimize their adverse effects, and preserve their potency. Currently, DDS significantly improves the availability of drugs in the body, enhances their absorption, regulates the release of drugs to maintain consistent levels, and targets specific cells to reduce side effects [2]. Nanotechnology-based DDS is a growing field in medicine delivery that addresses various challenges. These include enhancing bioavailability in the face of low solubility, addressing shortcomings in intestinal absorption mechanisms through degradation, improving delivery precision to target sites, enhancing therapeutic efficacy, minimizing side effects, and mitigating fluctuations in drug levels within the bloodstream [3]. NPs are a class of materials in nanotechnology, characterized by particle sizes ranging from 10 to 100 nm. These materials exhibit diverse structural dimensions, including zero- and one-dimensional configurations. NPs are composed of a core, shell, and surface layer that can be modified with various functional groups and possess unique properties. Their applications span numerous fields, with particular promise in the biomedical domain, including drug delivery, chemical and biological sensing, gas sensing, and carbon dioxide capture [4]. Various classes of nanomaterials have been used in the medical field. These include dendrimers, metallic NPs, carbon nanomaterials (such as nanorods, nanowires, tubes, fibers, and platelets) [5,6], magnetic nanomaterials, semiconductor nanomaterials [7], hydrogel [8], nanocomposites [9], biodegradable polymers, liposomes [10], and polymer nanocomposites [11]. Each class offers unique properties that can be harnessed for medical applications such as drug delivery, imaging, and tissue engineering (TE) [12,13]. NFs stand out among the different nanomaterials because they mimic the porous and fibrous structures in biological tissues, making them valuable for multifaceted applications [14]. NFs have garnered significant interest in delivering medicine due to their unique properties like mechanical characteristics of polymeric NFs, such as the modulus, shear modulus, and tensile strength, which improve as the fiber diameter decreases and are used to govern the behavior of cells and provide vigor to endure the forces of the cell cytoskeleton [15,16].

Biopolymers are emerging as promising candidates for the fabrication of NFs due to their biocompatibility, biodegradability, and versatility in various biomedical applications [17]. Ge and CH have gained significant attention among various biopolymers due to their unique and functional properties, which are essential for NF development. CH is an excellent option for biomedical applications such as TE scaffolds, wound dressings, and DDS due to its exceptional biocompatibility, anti-bacterial activity, and wound healing capabilities [18]. CH is produced by the deacetylation of chitin [19], while Ge, a denatured form of collagen derived from animal connective tissues, provides good biocompatibility and cell adhesion capabilities [20]. These properties are essential for TE and regenerative medicine. Owing to their structural resemblance to the extracellular matrix (ECM) [21], Ge-based NFs stimulate cellular connections and facilitate tissue regeneration. The tuneable mechanical characteristics of Ge-based NF can be controlled by modifying the electrospinning process settings. This allows the fabrication of scaffolds with customized stiffness and elasticity that resemble natural tissues. Furthermore, the versatility and application of Ge in the fabrication of controlled-release DDS and wound healing therapies are expanded by its susceptibility to chemical alterations and mixing with other polymers [22]. GE, a protein-rich in glycine, proline, and hydroxyproline, has various applications, such as in plasma extrusion, wound dressing, and adhesives, and as an absorbent pad during surgery [23]. According to research, it has been claimed that GE can activate macrophages, can exhibit a strong hemostatic effect, and does not induce antigenicity [24]. The present review explores additive preparation methods, including techniques such as electrospinning, force pinning, and template synthesis. Moreover, the review also focuses on electrospinning and discusses the process, recent advancements, and factors influencing the fiber structure. Additionally, the review covers polymer properties, the role of GE/CH in drug release, formulation strategies, and characterization techniques, focusing on fabrication using electrospun technology. In addition, applications in DDSs, orthopedics, wound healing, and regulatory considerations are addressed, along with challenges related to polymers.

## 2. Nanofibers

**Figure 1:**
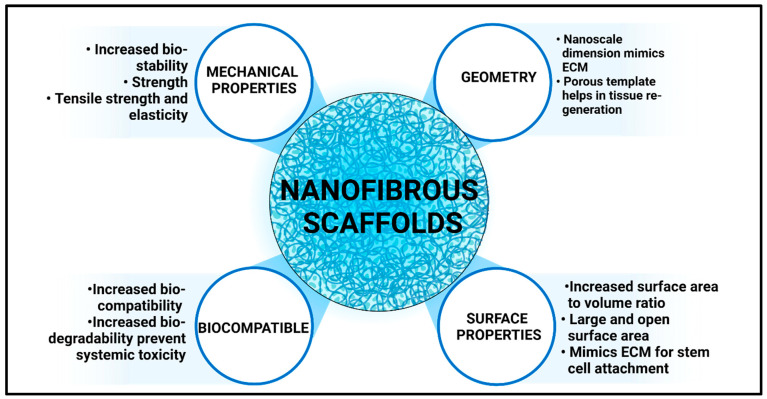
Illustration for ideal properties of NFs.

Nanofibers are ultrathin fibers, ranging from 1 to 1000 nm in diameter, produced using polymers. Although according to ASTM (2012) and ISO/ASTM standards, a nanomaterial is defined as having at least one dimension in the 1–100 nm range, many studies classify fibers with diameters exceeding 100 nm as nanofibers due to their nanoscale properties and functional advantages [25]. Using polymeric fibers and controlled-release administration methods, medications can be administered once or twice daily, enhancing patient compliance and preventing toxic plasma peaks resulting from frequent dosing of immediate-release formulations. These small NFs offer several benefits, such as exceptional stability, targeted delivery, high drug-loading capacity, increased surface area, reduced toxicity, enhanced mechanical properties, and suitability for delivering heat-sensitive drugs [26] (Figure 1). Electrospinning began over four centuries ago with William Gilbert’s work around 1600, pioneering this technique. Key developments included Louis Schwabe’s silk spinning method in 1845, Hughes and Chambers’ first carbon NF patent in 1889, and John Francis Cooley’s 1902 patent for the first electrospinning machine. In 1938, “Petryanov filters” were developed using electrospun fibers, and hollow graphitic carbon fibers were invented in 1952. Doshi and Reneker popularized “electrospinning” in 1995. This process is preferred for producing NFs with high surface-area-to-volume ratios and numerous pores [27].

### Nanofiber Fabrication Techniques

**Figure 2:**
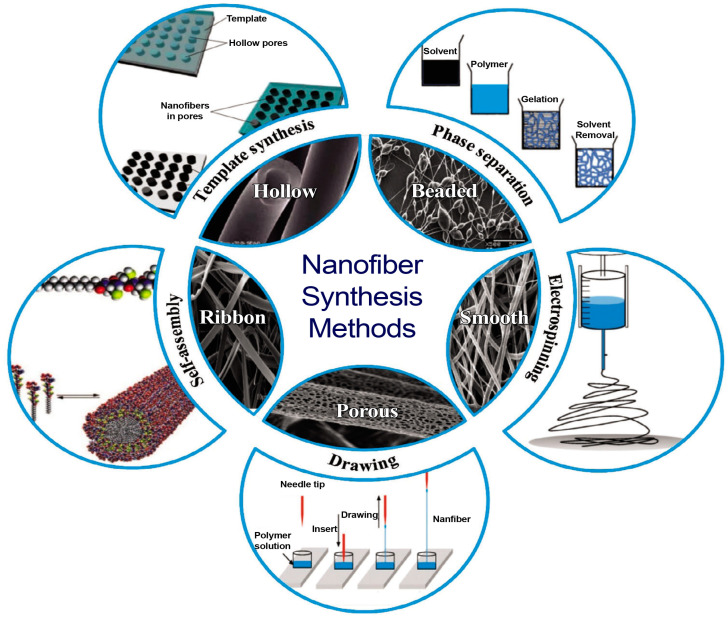
Illustration demonstrating comparative analysis of different NF fabrication techniques. Reproduced with permission from [32]. Copyright Clearance Center’s RightsLink service.

NFs are fabricated using various methods (Figure 2), each offering distinct advantages and applications. Electrospinning is the most widely used method whereas other notable techniques include phase separation, where a polymer solution undergoes gelation and phase extraction to form NFs, and template synthesis, which utilizes porous membranes to guide the formation of fibers. Also, self-assembly involves molecules’ spontaneous organization into fiber structures, although it typically requires complex procedures and has low productivity. Techniques for fabricating NFs are divided into several categories, including physical, chemical, and biological procedures, as well as top-down and bottom-up approaches. The top-down method reduces complicated materials to micro- or nanoscale particles, whereas the bottom-up method assembles small particles, usually between 1 and 10 nm, to form new bulk materials [28]. Physical techniques use mechanical energy or high-energy radiation to develop NFs. In contrast, chemical techniques, part of the bottom-up approach, depend on specific conditions to help atoms or ions combine to form clusters of single NPs. Environmentally friendly biological methods develop NFs by treating source materials with enzymes or microorganisms [29].

Electrospinning: It is a prominent technique that relies on parameters such as voltage, viscosity, solvent choice, and tip-to-collector distance. It is widely employed in the development of NFs from polymers and the incorporation of NPs. For example, polyurethane, polyacrylonitrile, and polyvinylidene fluoride yield NFs with diameters of approximately 150 nm, requiring a voltage of approximately 11 kV. Similarly, using acetone as a solvent and combining cellulose acetate with essential oils result in NFs that can be produced ranging between 700 and 1500 nm in size, necessitating a higher voltage of 120 kV [30]. Biologically generated NFs, such as eugenol and GE, are fabricated using acetic acid, ethanol, and water solvents. Their diameters range from 157 nm to 293 nm at a voltage of 13 kV. Moreover, NFs fabricated using GE produce a diameter of approximately 422 nm when mixed with polyethylene oxide, silica, and ciprofloxacin [31].

The drawing process can result in the development of fibers that are long and exhibit a diameter of several nanometers. Each NF is drawn using a micropipette during solvent evaporation and at the moment of solidification. Cooling and solvent evaporation are the two processes that cause freezing [32].

The template synthesis technique uses different solvents and templates, such as cellulose or a phenylalanine–phenylalanine–aspartic acid tripeptide, to produce NFs with regulated pore sizes and shapes.

Self-assembly: Small molecules are used as the fundamental building blocks in the self-assembly method [32].

Phase-separation-mediated NF growth demonstrates a separation of phase due to physical incompatibility. In this technique, a gel is prepared by lowering the temperature of the polymer solution developed by mixing a polymer material with an appropriate solvent. After producing the gel, it is immersed in another solvent that causes the gel to separate from the initial solvent, and in this way, the separation occurs between the two phases. The resulting polymer structures typically exhibit pores with a sponge-like morphology and spherical pores [32].

**Figure 3:**
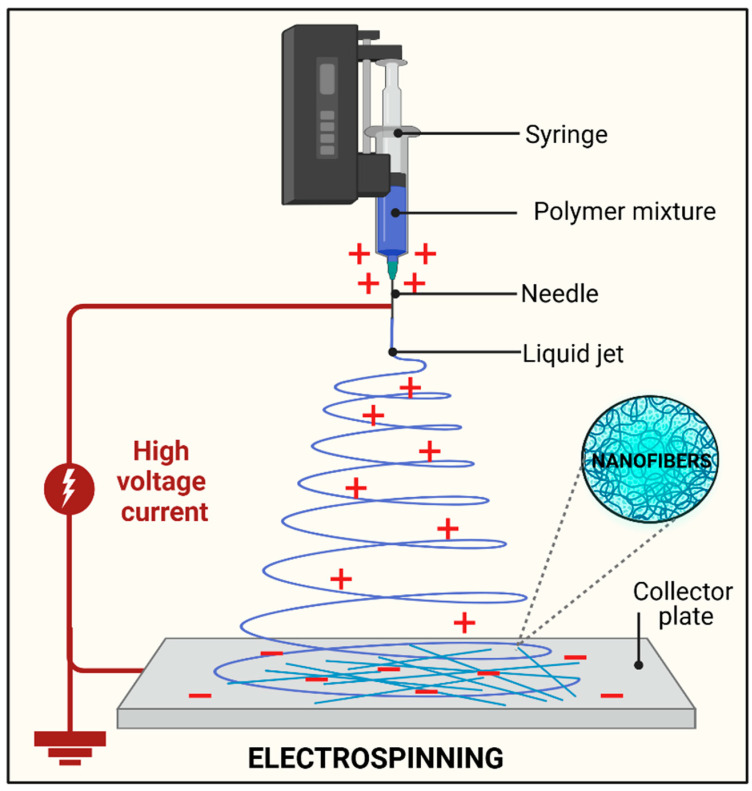
Illustration for fabrication of NFs using electrospinning.

Force spinning or centrifugal spinning, also known as rotary or rotational jet spinning, has been extensively developed and utilized in fabricating NFs. The primary distinction between force spinning and regular electrospinning is that the first uses a centrifugal force instead of an electric field, which is used in electrospinning. In this method, a liquid jet is ejected from the nozzle tip when the centrifugal force exceeds the surface tension and the spinning head containing the polymer solution reaches a crucial rotational speed. Subsequently, the jets are stretched, and the solvent evaporates, causing the deposition of NFs over the collector. The temperature, collecting system (e.g., collection distance), nozzle configuration, rotating speed of the heated structure, and other parameters determine the geometry and morphology of the centrifugally spun NFs [33], as shown in Figure 3. Many conductive and nonconductive materials can be spun using centrifugal spinning techniques. However, certain solid materials do not require chemical pretreatment before spinning after melting [34]. Centrifugal spinning eliminates the safety problems associated with electrospinning because it does not require high voltage. Moreover, force spinning helps to reduce typical electrospinning constraints, such as high electric field requirements, limited productivity, conductivity difficulties, and expensive production costs. Compared with electrospinning, centrifugal spinning yields NFs using polymers at higher concentrations, low solvent usage, and reduced production costs. Nevertheless, a notable drawback of centrifugal spinning is that fiber quality and productivity are profoundly influenced by the material properties and spinneret design [35]. Direct current and alternating current are two possible power source types. The electrospinning process can be broadly described as follows [36]: (i) charging the liquid droplet and developing a cone-shaped or Taylor cone-shaped jet, and the charged jet extends in a straight line; (ii) the application of a high-voltage electric field, ejection, and the formation of a polymer jet; (iii) thinning the jet in the presence of an electric field and producing an electrical bending instability, also called whipping instability; (iv) solidify and collect the jet as a solid fiber on a grounded collector [36].

CH and Ge NFs are versatile materials that can be used in various applications, including wound healing, drug delivery, tissue engineering, etc. Concise electrospinning is a process that involves applying a voltage to stretch polymer solutions into a fine fiber form. The flow rate and distance between the needle and collector are crucial parameters while fabricating fiber with uniformity. Additionally, environmental conditions such as temperature, humidity, and airflow also impact the process. Therefore, the proper control of these factors is essential for producing smooth, defect-free nanofibers, whereas proper temperature and humidity control are vital for consistency and reproducibility. Airflow around the setup affects fiber deposition patterns. Post-treatment and crosslinking are essential steps for stabilizing nanofibers, enhancing mechanical strength, water resistance, and durability [37].

CH is a rigid polysaccharide [38,39] that requires blending with flexible polymers like polyethylene oxide or polyvinyl alcohol to improve its spinnability and mechanical strength [11,40]. The critical electrospinning process involves parameters like polymer concentration, solution viscosity, conductivity, and environmental conditions. Moreover, the process involves optimizing the voltage applied and flow rate of the polymer solution to avoid bead formation and ensure smooth, continuous fibers [41]. CH, a polymeric biomaterial aligned with rigid D-glucosamine repeat units, is difficult to convert into a submicron-sized fibrous form due to its low solubility in water and organic solvents. However, modified chitosan demonstrates improvement in solubility when hydrogen bonds form water molecules, which are prevented by electrostatic repulsive interactions between positive ammonium groups. Acetic acid is a common pH adjustment reagent, and pure chitosan can be electrospun using a solvent with a high concentration. However, reducing pH using acids can make chitosan solutions more viscous, affecting spinnability. The electrospinning technique for chitosan NFs further faces challenges like limited solvent availability, ultimately affecting the quality and yield [42]. Aniseiei et al. fabricated CH-based NFs using HFIP as a solvent due to its low boiling point and ability to interrupt hydrogen bonding networks. Chitosan solutions were successfully electrospun at 0.4% concentration, producing amorphous nanofibers with a mean diameter of 42 ± 15 nm. The experimental results of the study revealed that fibers stick together under slightly acidic conditions, highlighting the challenge of managing solvent quantities during the electrospinning process [43].

Ge is another versatile polymeric material suitable for various applications [44]. However, their electrospinning process presents challenges due to their hydrophilic nature and tendency to dissolve or degrade in aqueous environments. Solution parameters, such as polymer concentration, molecular weight, solvent choice, and blend ratio, play a crucial role in the fabrication of nanofiber using Ge [45]. Ge is often combined with synthetic polymers or additives to improve fiber stability and mechanical performance. Blend ratios affect fiber properties, with higher proportions increasing bioactivity but reducing stability. Process parameters, such as applied voltage, flow rate, and needle-to-collector distance, also play a pivotal role in controlling fiber morphology and uniformity, while environmental factors, such as temperature, humidity, and airflow, influence the electrospinning process [45]. Moreover, the temperature affects the viscosity of the spinning solution and the evaporation rate of the solvent. In contrast, humidity plays a critical role in determining fiber morphology, with high humidity promoting the condensation of solvent vapors on the fiber surface, while controlled humidity ensures uniform and smooth fibers. Airflow around the electrospinning setup affects fiber deposition patterns and alignment. Post-spinning treatments, such as chemical crosslinking, are vital for enhancing the mechanical stability and water resistance of gelatin nanofibers. However, chemical crosslinkers may introduce cytotoxicity, requiring thorough removal of residual agents to maintain biocompatibility. Physical crosslinking methods, such as heat treatment or UV irradiation, offer residue-free alternatives but may compromise fiber morphology or mechanical properties [46].

## 3. Advancements in Electrospinning

Advancements in the fabrication of NFs through electrospinning technology have significantly enhanced the capabilities and applications of these materials, particularly in three-dimensional (3D) and four-dimensional (4D) cases using techniques such as chaotic electrospinning, which allows the development of multilayered structures with improved surface area and functionality, making them suitable for applications like energy storage area and biomedical scaffolds. Additionally, four-dimensional electrospinning incorporates time as a variable, enabling the fabrication of response to external stimuli, thus broadening their potential uses in innovative materials and DDS. Additionally, techniques such as near-field electrospinning have also emerged, which provide precise control over fiber deposition, allowing for the development of aligned fibers with specific topologies that enhance mechanical properties and cellular interactions. These innovations address traditional challenges in NF production, such as chaotic fiber deposition and low yield, and pave the way for more sophisticated applications in fields ranging from medicine to energy storage.

The electrospinning technique is now capable of more than two-dimensional fiber deposition owing to recent advancements. The development of 3D and 4D electrospinning methods has brought about revolutionary changes in several sectors, such as drug delivery [47], filtration [48], TE [48], and energy storage [49]. The engineering and science of sophisticated materials have been completely transformed using 3D printing. Rapid prototyping, often known as additive manufacturing technology or 3D printing, is a simple method for fabricating 3D products/materials that have been computer-designed. The software opens the door to innovation and intricate product design with ground-breaking functionality that will completely change the manufacturing landscape. Nonetheless, two significant drawbacks of 3D printing are its limited resolution and sluggish pace of production [50]. The term “4D printing” refers to the process of 3D printing, which exhibits the ability to alter shape over time or in response to external stimuli. These shape-changing materials have applications in medication delivery, biomedical devices, soft robotic systems, and intelligent textiles. They can also adapt to changing environmental factors, including heat, light, moisture, and pH. This revolutionary new technology will make the goods of the future possible, although 4D printing at the nanoscale has not yet been investigated [51]. Electrospinning is a highly adaptable method that can quickly fabricate 3D/4D printing solutions at the nanoscale by processing melts, solutions, or suspensions into continuous NFs using a high voltage. A revolutionary nano-printing technique called 3D/4D electrospinning combines the benefits of electrospinning with 3D printing technology [52]. Additionally, 3D/4D structures at the nanoscale provide new prospects for material development. Fibers are deposited through three-dimensional structures using several nozzles or platforms in 3D electrospinning. This method makes it possible to precisely regulate the orientation and distribution of fibers while fabricating intricate scaffolds. However, 4D electrospinning adds a temporal dimension that permits dynamic modifications to the shape of the fiber or other characteristics after deposition. This potential invents new opportunities for developing responsive fabrics, programmed medication-release systems, and shape-memory materials. Enhancement of functionality and adaptation to different physiological or environmental conditions is possible with 4D electrospinning through the incorporation of stimuli-responsive materials or controlled degradation processes [53].

## 4. Biocompatible and Biodegradable Polymers for Fabrication of Nanofibers

**Figure 4:**
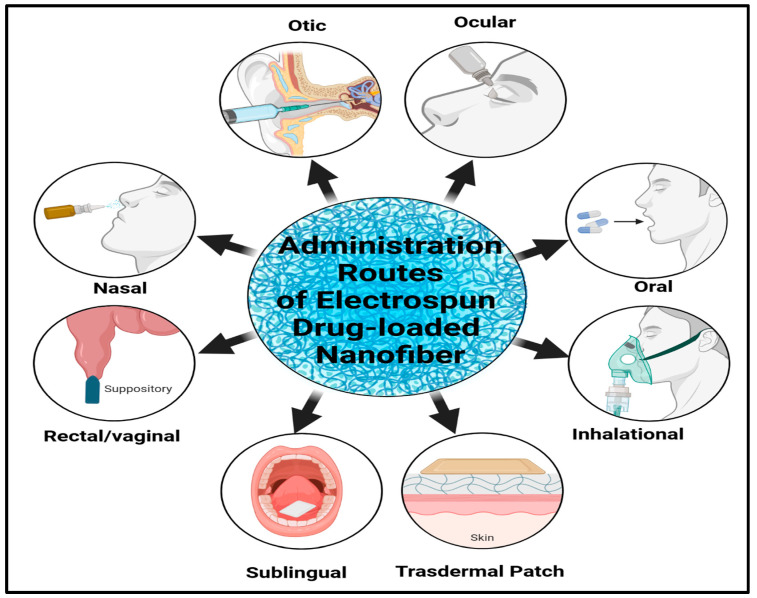
Common administration routes of drug-loaded GE/CH nanofibers.

Biocompatible and biodegradable polymers play a crucial role in the fabrication of NFS, which are increasingly utilized in various applications, such as TE, drug delivery, and environmental remediation, as they interact harmoniously with biological systems. Different polymers have been employed in the fabrication of NFs using electrospinning. Natural polymers are collagen, alginate, Ge, CH, etc., whereas synthetic polymers, including polycaprolactone (PCL), polylactic acid [54], poly(lactic-co-glycolic acid) (PLGA), etc., offer advantages such as tuneable mechanical properties and ease of processing, making them suitable for diverse applications (Figure 4). Their selection is based on the type of targeted action and the method of preparation. Herein, we only focus on using natural polymers such as GE and GE for the fabrication of NFs, as they are biocompatible and quickly degrade when they come in contact with biological tissue.

### 4.1. Gelatin

**Figure 5:**
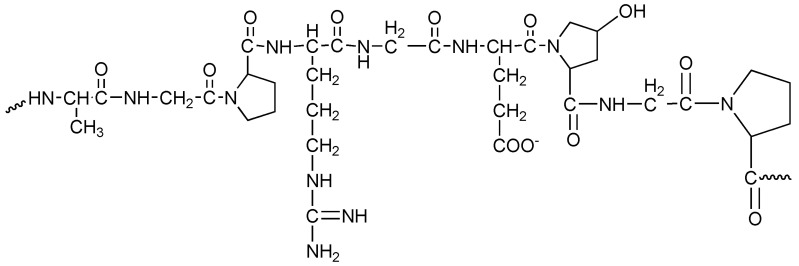
Structure of gelatin.

GE (Figure 5) is a water-soluble biopolymer versatile protein produced through collagen hydrolysis. Its biodegradability, bio-affinity, formability, and cost effectiveness make it a valuable material in various fields, including the biomedical and environmental fields [55]. GE can be facilitated in various shapes, including NPs, microparticles, films, hydrogels, and NFs. Owing to their low electrospinnability, suitable solvents like 2,2,2-trifluoroethanol, acetic acid, and formic acid are used [56]. Mixtures of GE and synthetic and natural polymers offer desirable biocompatibility and bio-mechanical characteristics. Electrospun Ge-NFs are ideal for drug delivery, wound dressings, and cell and tissue culture. GE is preliminarily found in plant connective tissues and has a significant role in preserving the integrity of connective tissues, including bones, corneas, cartilage, and tendons. Approximately 80–90% of the collagen in the body is made up of the three most common forms: type I, type II, and type III [57]. Three helical polypeptide chains, each measuring around 1.5 nm in diameter and 300 nm in length, make up collagen type I [58]. Two α-chains and one β-chain form the typical triple-helix structure of type I collagen are the molecules in the middle regions, and the polypeptide architectures of the three spiral chains of collagen have limited immunogenicity and antigenicity [59]. The limited antigenicity of collagen has led to a significant decrease in its use in biological applications and its involvement in the manufacturing of NFs [60].

### 4.2. Chitosan

**Figure 6:**
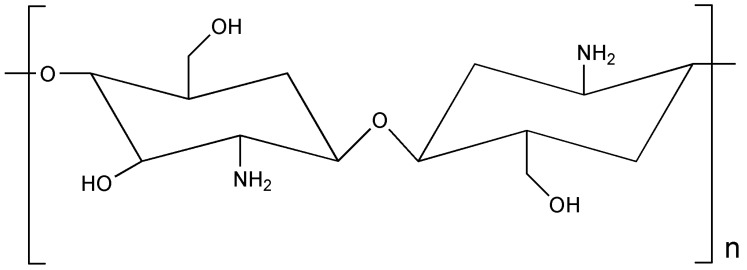
Structure of chitosan.

CH (Figure 6), which is thought to be the second most abundant biopolymer after cellulose, is obtained from natural sources that can be fungal, aquatic, or terrestrial. Chitin is the starting material obtained after chemical and enzymatic treatments, and GE is obtained by deacetylating chitin [61]. The semi-crystalline substance known as GE is composed of N-acetyl-d-glucosamine residues. GE molecules are composed of hydroxyl (–OH) and amino (–NH_2_) groups [62]. This particular polymer is cationic, which aids in constructing ECM components by drawing negatively charged ions. It also exhibits biopotential activities, improves mechanical strength, and strengthens bonds by causing structural changes. GE, a cationic biopolymer, is problematic to electrospin because of its rigid D-glucosamine repeat unit, high crystallinity, and hydrogen bonding ability. For electrospun defect-free fibers, the polymer concentration must be at least 2 to 2.5 times the entanglement concentration [63]. However, even moderate concentrations can become too viscous to overcome the electric field, making electrospinning impossible. The rheology of the solutions also affects the electrospinning process. To improve electrospunability, researchers have blended GE with other polymers [64]. A comparative feature of GE and CH is presented in Table 1, collected from various previously published data [65,66,67,68,69,70,71,72,73,74,75].

## 5. Formulation Strategies for Polymer-Based Electrospun Drug Delivery Systems

Polymer-based NFs are widely used owing to their unique properties, such as a high surface-area-to-volume ratio, tuneable porosity, and ability to encapsulate a wide range of therapeutic applications [76]. To ensure controlled release, improved stability, and therapeutic efficacy, formulating an effective NF-based DDS involves intricate formulation strategies such as polymer selection, a drug encapsulation method, an optimization process, and material parameters [77].

Synthetic polymers (PLGA and PCL) and natural polymers (GE and CH) are used in controlled-release applications owing to their tuneable degradation rate, safety profile, compatibility with surfactants or co-solvents, and TE applications [78]. Moreover, the polymer ratio is another crucial aspect to be considered, as changing the concentration of polymers may affect the NF structure and release pattern. Vu et al. successfully fabricated polyvinyl alcohol (PVA)–CH NFs to understand the effect of polymers using a solution containing 5% PVA, 5% CH, and 60% CH_3_COOH, with diameters ranging from 77 to 292 nm and an average diameter of 153 nm, suitable for drug integration [79].

The second parameter is drug incorporation: drugs or bioactive compounds can be added to the polymer either in melted or in solution form using various techniques before electrospinning. Drugs are dispersed throughout the polymer solution by solvent mixing, sonication, or stirring for physical encapsulation. Both hydrophilic and hydrophobic medications can be used with this technique; yet, the NFs may distribute pharmaceuticals unevenly [79]. Chemical conjugation, on the other hand, entails covalently joining medications or functional groups to polymer chains to improve stability and allow regulated release. This technique guarantees precise control over drug-loading and release kinetics; compatibility between drug and polymer chemistry is necessary [80]. Combining polymers of different features using blend electrospinning can produce synergistic advantages, including improved drug release patterns, biodegradation, and mechanical strength. Additionally, the polymer ratio influences the physical and chemical interactions between the drug molecules and the polymer matrix, which substantially impacts the entrapment efficiency of the electrospun NFs [81]. The polymer’s solubility, the spinning solution’s viscosity, electrospinning parameters, and the compatibility of the drug with the polymers all affect the entrapment efficiency. To achieve maximum drug loading, entrapment efficiency can be optimized by combining polymers with diverse properties (e.g., hydrophilic and hydrophobic). Hydrophilic polymers have been observed to interact favorably with hydrophilic medicines through hydrogen bonding or electrostatic interactions, leading to improved drug encapsulation inside the NF matrix. On the other hand, owing to enhanced compatibility and less drug outflow during electrospinning, hydrophobic medicines may show better entrapment efficiencies when electrospun with hydrophobic polymers [81,82]. The third parameter is controlled release and the release behavior of drugs from electrospun NFs, which is closely intertwined with the entrapment efficiency and polymer ratio. A higher entrapment efficiency typically results in more significant drug loading within the NFs, directly influencing the initial burst release and sustained release kinetics over time [83]. The polymer ratio controls factors such as the drug encapsulation efficiency, release kinetics, and drug liberation mechanism, all of which are directly related to the release of pharmaceuticals from the electrospun NFs. Furthermore, the drug release profile is regulated by modifying the polymer composition, including the polymer degradation rate, swelling behavior, and diffusion paths within the NF matrix [84].

## 6. Characterization Techniques Involved with Polymeric Electrospun Nanofibers

The physical, chemical, and structural characteristics of polymeric electrospun NFs must be evaluated to determine their applicability in various applications, including filtration, drug delivery, and TE. As described below, the electrospun nanofibers are characterized through primary advanced instrumental techniques. 

### 6.1. Microscopic Analysis for Fiber Morphology Assessment

**Figure 7:**
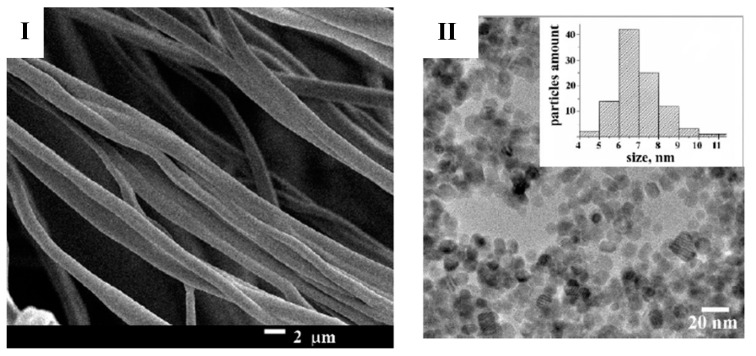
Morphological illustration of GE NFs (**I**) and TEM microscopy image of ZnO-NP-coated NFs with particle size distribution (**II**). Reproduced with permission from [86] under CCBY.

Scanning electron microscopy [SEM] and transmission electron microscopy [TEM] are used to visualize the morphology and surface topology of NFs. SEM allows the examination of cross-sectional views and enables analyses of the fiber structure, layering, and defects, whereas TEM provides high-resolution images and detailed imaging of NF structures at the nanoscale to elucidate the interaction between polymers and drug molecules within the NF matrix [85]. A microscopic examination using SEM for NF illustration is presented in Figure 7.

### 6.2. Evaluation of Mechanical Properties of Electrospun Nanofibers

The mechanical properties such as tensile strength, elasticity, and flexibility of the NFs are assessed using the tensile testing method following AST standards. This test measures the force required to stretch or deform the NFs, providing quantitative data on mechanical behavior under physiological or operational conditions, as these are necessary for TE scaffolds and wound dressings [87].

### 6.3. Material Interactions Through Spectroscopic Techniques

Various spectral/functional techniques have been employed to study the interactions between polymers and drug molecules between ECM and NFs, while FTIR has been used to analyze the chemical and molecular structures. FTIR spectra generally detect changes in the polymer conformation and crystallinity induced during electrospinning [88,89]. On the other hand, X-ray diffraction is used to investigate the crystalline structure and phase transition of the NFs. It provides information on the size, direction, and degree of crystallinity of crystallites by identifying the crystalline peaks corresponding to polymer chains or embedded compounds. XRD studies enabled understanding of the changes in NF structure under mechanical stress or environmental circumstances, managing drug release processes driven by crystalline domains, and optimizing polymer blends [90]. A thermogravimetric analysis and differential scanning calorimetry are two thermal characterization methods used to assess the stability, phase transitions, and thermal behavior of electrospun NFs. Differential scanning calorimetry sheds light on the mobility of polymer chains and thermal stability by measuring the variations in heat flow caused by melting, crystallization, or glass transition temperatures. By calculating the weight loss and breakdown temperatures of NFs, the thermogravimetric analysis evaluates their degradation kinetics and appropriateness for specific uses that require regulated or heat-resistant degradation [91].

## 7. Characterization Techniques Involved with Polymeric Electrospun Nanofibers

Biomimetic applications of polymer-based electrospun NF materials, utilizing nature-inspired design principles, offer advanced functional materials with unique properties, such as a high surface-area-to-volume ratio and mechanical flexibility.

### 7.1. Transdermal Drug Delivery Systems

**Figure 8:**
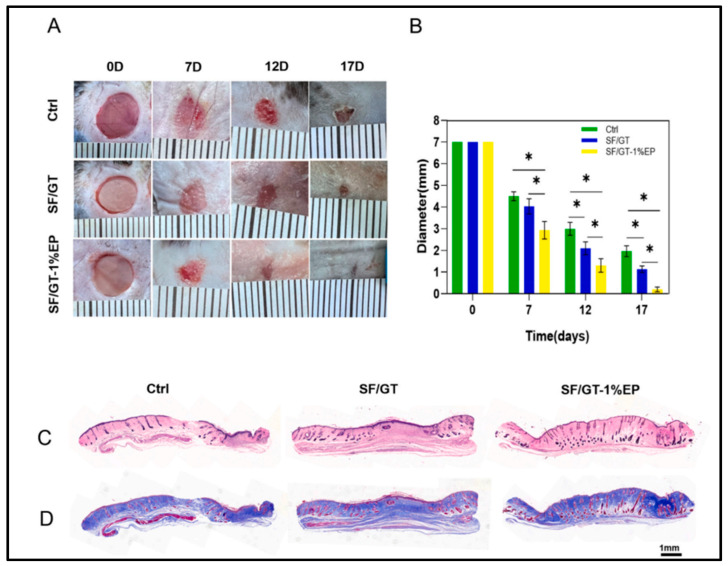
In vivo wound healing results. Macroscopic photographs of wounds at 0, 7, 12, and 17 days after a full-thickness skin wound was created (**A**). Wound diameters (0, 7, 12, and 17 days) of different groups (control, SF/GT, and SF/GT-1%-propolis extract) (**B**). Histology analyses of the wound area sections at different groups 17 days after the operation (control, SF/GT, and SF/GT-1%-propolis extract) (**C**,**D**). Reproduced with permission from [93] under CCBY.

Transdermal DDS (TDDS) offers a non-invasive, patient-friendly method of delivering therapeutic agents through the skin for localized or systemic effects. This research represents a significant gap in pharmaceutical technology [92]. Owing to their distinct structural and functional characteristics, NFs, especially electrospun NFs, have emerged as intriguing platforms for improving TDDS. The use of NFs in TDDS is examined in this article, which also discusses current developments, difficulties, benefits, and possible future paths. To produce systemic circulation or specific local effects, transdermal medication delivery entails the introduction of medicinal substances through the stratum corneum, the outermost layer of the skin. The inherent impermeability of the skin restricts the administration of hydrophilic medications and large molecules, which is why conventional transdermal patches and topical formulations rely on passive diffusion through the skin barrier. Overcoming these obstacles while preserving therapeutic efficacy and patient compliance is a significant problem in transdermal drug delivery. NFs are widely used to deliver drugs directly to the skin surface in dermatological conditions such as wound healing, tissue regeneration, psoriasis, and other fungal infections [92]. NF patches are commonly used to deliver hormones or vaccines to the skin. Du et al. fabricated propolis extract-enriched silk fibroin (SF)/Ge NFs as wound dressings. These findings revealed that both cytocompatibility and hemocompatibility were suitable for the SF/Ge-1%-propolis extract. Furthermore, it has the potential to enhance fibroblast cell motility significantly. The SF/Ge-1%-propolis extract was applied to a mouse model of full-thickness skin defects (Figure 8) [93].

Goudarzi et al. fabricated GE-PVA NFs for skin TE, and their findings revealed excellent cell viability and cell attachment of NFs for mouse fibroblast cells according to the MTT assay, which indicated that the use of both GE and GE complexes exhibit potent activity [94]. Doostmohammadi et al. used a histopathological analysis to examine the in vivo wound healing potential of tellurium NPs embedded in PCL and Ge-NFs (electrospun). This study was conducted on wounds in Wistar rats. Scaffolds incorporating NPs demonstrated the highest healing, scoring 15 out of 19. Furthermore, the scaffold reduced edema and inflammation at the injury site and had favorable effects on collagen horizontalization and synthesis in a dose-dependent manner. Their healing action was verified by the data, which can be helpful for wound dressing [95].

### 7.2. Orthopedic and Wound Healing Applications

**Figure 9:**
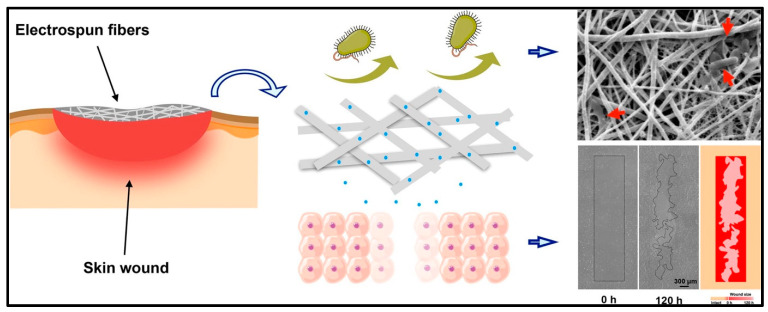
Illustration for NFs and their role in wound healing. Reproduced with permission from [108] under Rightlinks Copyright Clearance Center.

**Figure 10:**
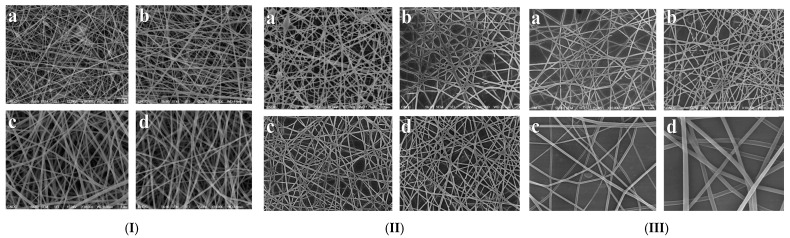
Scan electron microscopy images of PVA, GE, and their blend at ratio of (**I-a**) 0/10 [109], (**I-b**) 2/8, (**I-c**) 6/4, and (**I-d**) 8/2. Scan electron microscopy images of PVA-GE blend at ratio of 2/8 with applied voltages of (**II-a**) 18 kV, (**II-b**) 20 kV, (**II-c**) 22 kV, and (**II-d**) 24 kV at tip–target distance of 10 cm. Scan electron microscopy images of PVA-GE blend at ratio of 2/8 showing variation in diameter distribution with tip–target distance at (**III-a**) 7 cm, (**III-b**) 10 cm, (**III-c**) 15 cm, and (**III-d**) 20 cm at 22 kV. Reproduced with permission from [106] under Rightlinks Copyright Clearance Center.

**Figure 11:**
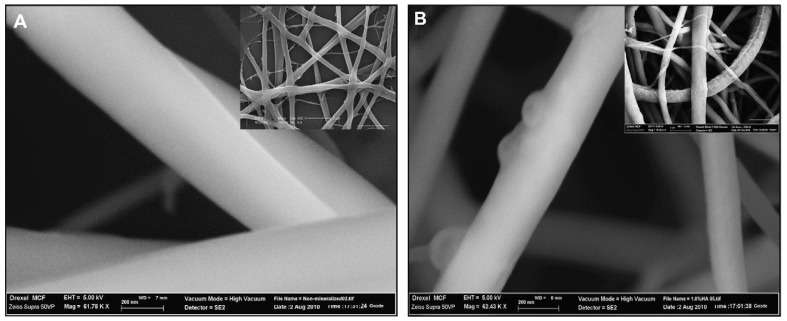
Scan electron image of 0.1% genipin crosslinked with chitosan electrospun scaffold mats: 0.1% genipin crosslinked with 1.0% hydroxyapatite (**A**) and 0.1% genipin crosslinked with 7.0% CH (**B**) at scale bar of 200 nm. Reproduced with permission from [107] under Rightlinks Copyright Clearance Center.

Originally, wound dressings and fracture healing were fabricated using natural substances such as plant fibers and animal fats, which were used to cover the injured area. This has progressed to the point where healing applicants can now be made from artificial materials using various cutting-edge technologies. Enhanced antimicrobial properties, quick hemostasis, and tissue repair are essential for contemporary wound healing (Figure 9) and other orthopedic treatments. Wound healing has the advantage of developing nanofibrous membranes with fast hemostasis and anti-bacterial properties using electrospinning techniques. Anti-bacterial NFs have been prepared using conventional anti-bacterial ingredients, such as metal oxide NPs, triclosan, antibiotics, biguanides, quaternary ammonium compounds, and silver NPs, using the electrospinning method for fast wound healing with less toxicity and side effects [96]. In clinical studies, diabetic foot ulcers have been identified as a prevalent and severe form of chronic wounds. Diabetic feet can result from ischemia, neuropathy, or neurobiochemical illnesses. A diabetic foot causes ulcers and infections or necessitates foot amputation [97]. By 2030, diabetes is expected to affect 552 million people globally, and 25% of them are likely to develop foot ulcers [98]. Castings and orthopedics are more expensive than patient compliance. Fiber scaffolds fabricated by electrospinning devices can provide structural and morphological clues for the adhesion of various types of cells in wounds and serve as templates for wound tissue regeneration. Microstructures similar to ECM proteins and electrospun fiber scaffolds play an essential role in maintaining cell growth and infiltration and are, therefore, suitable for use in TE [99]. Polymeric electrospun NFs are among the most thoroughly studied nanostructures for orthopedic TE applications. The interconnected porous structure of nanofibrous scaffolds offers a large surface area for cell adhesion and room for the passage of nutrients [100]. In addition to providing mechanical support, applying NFs as delivery systems for bioactive compounds at the wound site offers two of three domains to initiate and accelerate wound healing, which may lead to effective tissue repair or regeneration [101]. Electrospun NFs can reduce tissue reactivity, reduce inflammatory responses, and provide a native habitat for cells at the wound site. They also resemble collagen fibers in the ECM [102]. Poly(ethylene oxide), used for a variety of biomedical applications such as cartilage TE and wound dressing, can facilitate the electrospinning of GE in aqueous solvents and eliminate the possible toxic effect of organic solvent residues in the final electrospun mat [103]. The effectiveness of polymeric NF scaffolds in promoting osteoblast adhesion and proliferation was shown by Li et al., who demonstrated their capacity to direct the orientation of cell cytoskeletal proteins and offer a large surface area for cell adhesion and proliferation [104]. Chu et al. reported a hepatocyte nanofibrous scaffold composed of PCL–ethyl ethylene phosphate containing a galactose ligand, which can aid cell attachment, spheroid formation, and functional maintenance of hepatocytes [105]. In another study, Linn and co-workers fabricated NFs using alone PVA, and GE, and a blend of PVA with Ge for the TE, considering processing parameters such as the concentration of polymers, electrical field, and tip-to-collector distance. The results in NF diameter via morphology for alone PVA, and GE, and a blend of PVA with Ge ranged between 50 and 150 nm. At the same time, an X-ray diffraction analysis demonstrated the existence of the crystalline nature of NFs. Meanwhile, thermal and tensile strength tests suggested improved physical properties without significant chemical interaction during processing, suggesting great promise for use in TE applications [106] (Figure 10). In a study by electrospun hydroxyapatite-containing CH-NFs crosslinked with genipin for bone, TE was tested for physical structure and mechanical properties with a consideration that NFs’ composite may represent a similar microenvironment with the promotion of osteoblast differentiation and maturation. The resulting NFs showed a gain in diameter from 227 nm to 335 after genipin was added in CH-NFs with remarkable mechanical strength via Young’s modulus of 142 Mpa, similar to natural periosteum. Moreover, the expression and enzymatic activity of alkaline phosphates were 2.4-fold higher for genipin-incorporated CH-NFs in cell culture, compared to CH NFs alone, suggesting that crosslinking CH with hydroxyapatite facilitates the proliferation, differentiation, and maturation of osteoblast-like cells [107] (Figure 11).

### 7.3. Central Nervous System-Targeted Drug Delivery

Traumatic and vascular injuries to the central nervous system are major concerns for international health. The delivery of medications into the brain for the treatment of central nervous system (CNS) disorders and their repercussions, including encephalitis, multiple sclerosis, and Alzheimer’s disease, has become extremely difficult due to permeable barriers. Current pharmacological therapies are ineffective for treating CNS damage. Therefore, the use of nanocarrier-based drug delivery has become more popular as a novel and promising approach to neurological protection and treatment as a result of developments in nanotechnology and medicine [110]. Electrospinning offers the ability to adjust the micro/nano-architecture regarding fiber diameter, surface porosity, and orientation through process parameter control. Electrospun fiber scaffolds are a biomaterial technique that can direct axonal regeneration through spinal cord injury. When an electrospun fiber scaffold is implanted following spinal cord injury, it will change the phenotype of cells to best support functional recovery and have physical properties that allow for quick, targeted tissue regeneration. The behavior of CNS cells in vitro and in vivo is significantly influenced by fiber diameter, alignment, and density [111]. Dose, control, and site specificity are necessary to develop a revolutionary medication delivery system. These systems have been created using a variety of physical, chemical, mechanical, and biological signals, among which light has been used mainly in the last ten years. Peripheral nerve injury, spinal cord injury, and traumatic brain injury are examples of nervous system injuries that pose serious risks to patient health [112]. Polymeric electrospun materials are adaptable to various treatments, including proteins and small compounds. This makes it possible to implement customized therapies to reduce inflammation and hasten neuro-regeneration at the injury site [113].

Chemotherapeutics can be delivered to the tumor site at a high concentration and with prolonged release via electrospun NFs, reducing systemic exposure and toxicity. Aligned electrospun fibers can replicate the topographical structures of the tumor microenvironment encountered by invasive GBM cells in vitro, which can forecast cell activity in vivo and provide non-invasive treatment methods for malignant gliomas [114]. Bini et al. attempted to develop porous nanofibrous scaffolds using electrospinning. This method forms nanometer-diameter polymer fibers by applying a strong electric field to a stream of polymer fluid. Their study predicted the orientation and proliferation of nerve stem cells on PLGA, suggesting that it is a viable substrate for nerve tissue creation [115]. Yang developed brain and spinal cord neurites, especially those derived from in vitro-cultured neurons, guided in their interaction using two-dimensional nanofibrous scaffolds. They employed 3D nanofibrous scaffolds in brain and spinal cord injury models, both in vitro and in vivo, to create a favorable microenvironment for cell adhesion and contact guidance or structural bridges for axons to govern reconnection. They rebuild glial circuitry over the “lesion gaps” left by CNS injuries and direct neurite outgrowth, two potential uses for nanofibrous scaffolds. They are hypothesized to be composed of biodegradable and biocompatible materials [116]. Electrospun nanofibrous assemblies exhibit distinctive characteristics, including an elevated specific surface area, high porosity, and structural resemblance to the extracellular matrix. Owing to these qualities, they are beneficial in many domains, such as medication release and TE [117].

### 7.4. Vaginal-Targeted Drug Delivery

**Figure 12:**
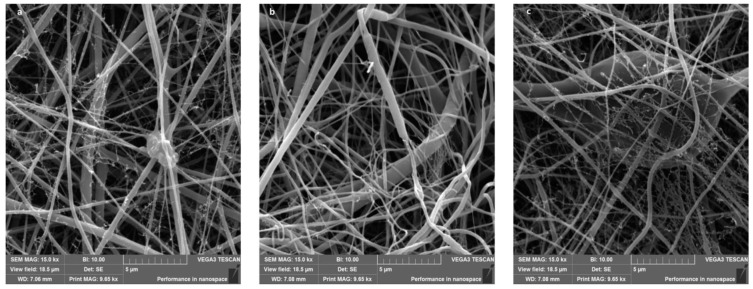
Scan electron microscopy image of Ge-PCL NFs fortified with *Myrtle berries* fruit (**a**), seeds (**b**), and leaves (**c**). Reproduced with permission from [123] under CCBY-4.0.

Drug-loaded electrospun NFs represent a novel approach to drug delivery via the vaginal mucosa, which may prove advantageous in situations where local drug delivery is necessary. The versatility of these NFs, high loading capacity, high mucoadhesive strength, typical softness, targeted drug delivery in the vagina, decreased toxicity to other organs, and lack of sharp corners that suit lesions with easy-to-use properties contribute to their benefits [118]. The ability of NFs to administer drugs directly to the vaginal mucosa is a noteworthy benefit. This feature is helpful in the treatment of disorders, such as infections, menstrual problems, and vaginal atrophy. By reducing systemic drug requirements, this localized therapy method lowers the risk of systemic adverse effects and improves the therapeutic outcomes. The capacity of NF-based systems to contour the vaginal membrane and provide effective action is another benefit [119]. Vidyavaridhi et al. fabricated luliconazole-loaded mucoadhesive PCL-GE NFs for anticandidal activity. The findings showed that the Fickian release mechanism allowed the release of luliconazole from LCZ-loaded NFs. When luliconazole was entrapped in NFs, as opposed to a drug suspension, penetration in the vaginal mucosa increased. Anticandidal action was demonstrated by in silico activity and microbiological assays [120]. Nematpur et al. fabricated clotrimazole-loaded NF films for vaginitis, and comparative studies were performed to assess their antifungal activity. This finding reveals that clotrimazole-loaded NFs exhibit higher antifungal activity than films [121]. Ball et al. developed a hollow tube-shaped NF suitable for a vaginal tampon applicator using a two-axis mandrel electrospinning rig for fiber collection [122]. Ge-PCL smooth NFs (Figure 12) fortified with *Myrtle berries*, which are a plethora of bioactive reservoirs and exhibit antioxidant, antiseptic, and anti-inflammatory activity, tested against a series of microbial flora showed significant capability in counteracting that microbial proliferation, suggesting the potential of *Myrtle berries* for future vaginal infection management [123].

The human immunodeficiency virus (HIV) still affects the health of millions of people worldwide despite numerous advancements in modern medicine, and significant efforts have been made to develop strategies to either prevent infection or remove the virus after it has already occurred. Antiviral medications are easily incorporated within electrospun cellulose acetate phthalate fibers during the electrospinning process and can be used as a preventative measure against HIV [124]. In a small-scale study, Agrawal et al. examined the therapeutic effectiveness of electrospun NFs loaded with cisplatin and a GE composite for the local treatment of cervical tumors in mice released for up to one month. When compared to ordinary medication, the prepared NFs demonstrated a lower percentage of cell survival, stronger mucoadhesive strength, and superior antitumor activity [125]. Herpes simplex virus infections caused by HSV-1 (oral herpes) and HSV-2 (genital herpes) were examined against electrospun NFs. This was achieved by encapsulating acyclovir in polymeric NFs for controlled-release delivery via intravaginal delivery [126]. The production of NFs with desired shapes provides an advantage for easy vaginal administration [127].

### 7.5. Ocular-Targeted Drug Delivery

**Figure 13:**
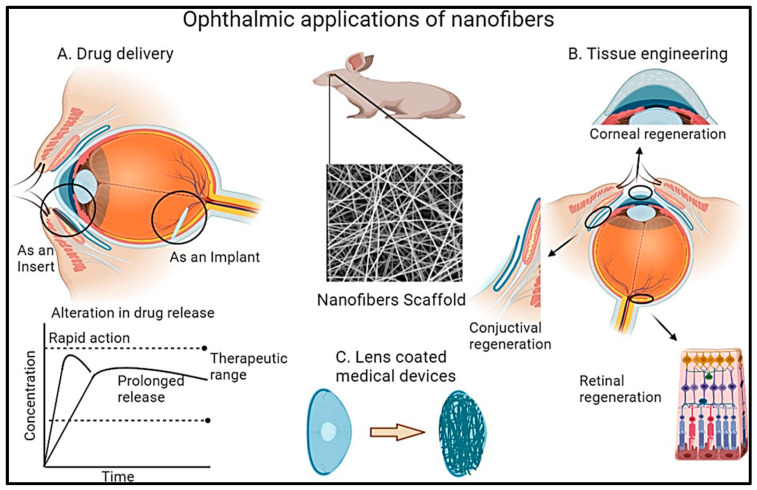
Illustration of ophthalmic applications of NFs.

**Figure 14:**
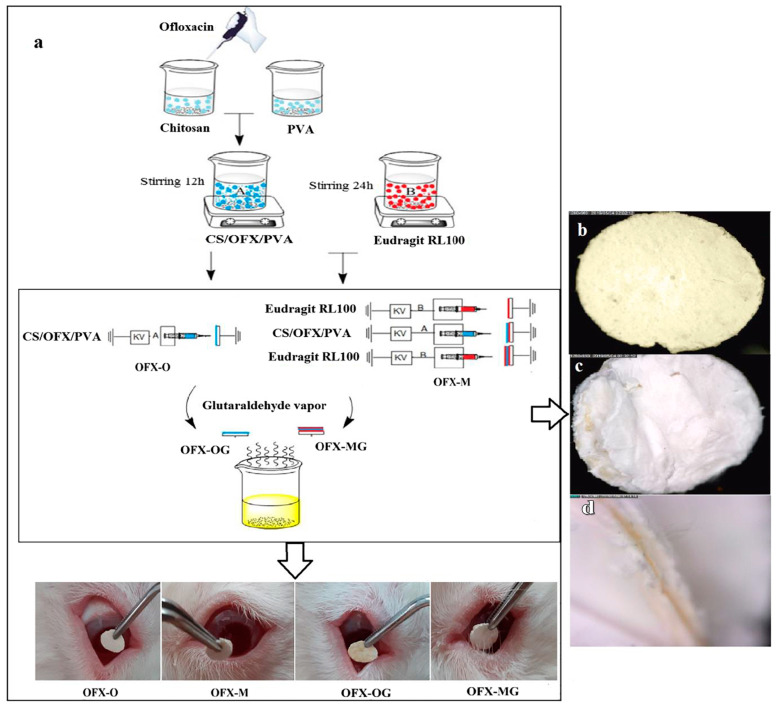
Illustration demonstrating fabrication of modified electrospun-based NFs (**a**) for enhanced ocular residence of ofloxacin using CH/PVA blends (**b**–**d**) as hydrophilic composition and Eudragit RL100/PVA crosslinked using glutaraldehyde for management of infectious conjunctivitis. Reproduced with permission from [131] under CCBY-4.0.

Since their inception as DDS, NFs have been utilized for ocular drug targeting. The materials employed in their fabrication enhance drug penetration and prolong the contact time with ocular tissues, demonstrating excellent biocompatibility. Furthermore, their extended-release profile decreases the dosing frequency [127]. Researchers have demonstrated the effective utilization of NF-incorporated drugs for the treatment of various ocular diseases and disorders (Figure 13). Cejkova et al. developed and utilized cyclosporine A NFs for treating alkali-injured corneas and demonstrated superior efficacy in suppressing inflammation and neovascularization compared with eye drops. The prolonged contact time of drug-loaded NFs with the injured eye has been suggested to be a key factor for their enhanced effectiveness [128]. Similarly, Gagandeep et al. reported the treatment of glaucoma with NFs of timolol maleate and dorzolamide hydrochloride, which showed a significant reduction in intraocular pressure compared to commercial eye drops [129]. In another study, Hsu et al. developed polycaprolactone NFs and microbeads for the extended release of dexamethasone, aiming to reduce the frequency of administration and associated side effects [130]. These studies illustrate the potential of NF-based DDS to improve the treatment outcomes for various eye disorders. Mirzaeei and co-workers developed modified electrospun-based NFs for enhanced ocular residence of ofloxacin using a CH/PVA blend as a hydrophilic composition and Eudragit RL100/PVA crosslinked using glutaraldehyde for the management of infectious conjunctivitis. The average diameter obtained for both types of NFs was around 123 nm and 159 nm when NFs were fortified with ofloxacin. The release content of ofloxacin from NFs was tested for antimicrobial potency against *Staphylococcus aureus* and *Escherichia coli*, demonstrating good activity. Moreover, in vivo studies suggested that NF inserts released the drug for 96 h with AUC_0-96_ of 9–20-fold, which was higher compared to that of the alone ofloxacin drug solution, suggesting the overall utility of NFs in ocular DDS (Figure 14) [131]. In another study, electrospun NFs of Ge crosslinked with hyaluronic acid through vapor of glutaraldehyde were fabricated for delivering human corneal mesenchymal stromal cells for the management of deep corneal injuries. The developed NFs exhibited excellent mechanics and transmittance with the improved proliferation of corneal mesenchymal stromal cells through a scratch migration assay after 48 h. Moreover, corneal wound healing and stromal α-smooth muscle actin lowered expression compared to those untreated keratectomy and corneal mesenchymal stromal cell groups, suggesting the therapeutic potential of NFs for the stromal regeneration and management of deep corneal defects [132].

## 8. Challenges and Key Considerations in the Fabrication of Nanofibers

Below are important factors affecting the shape and structure of fibers in the context of biopolymer-based nanofibers, as well as information on how they are prepared and manufactured additively.

Properties of the polymer solution: The viscosity, concentration, solvent type, and molecular weight of the polymer solution have a significant impact on the morphology of the fiber. Thicker fibers are often produced at higher polymer concentrations and viscosities, whereas finer fibers are produced at lower concentrations. Fiber diameter and bead formation are influenced by solvent volatility; smoother fibers usually result from slower evaporation rates [133].

Electric field parameters: In electrospinning, variables such as the voltage, flow rate, and separation between the spinneret and collector are essential. Higher flow rates result in bead production, whereas higher voltages produce more stretching and thinner fibers. The fiber diameter and alignment are influenced by the distance between the spinneret and collector; closer distances result in better-aligned fibers [134].

Environmental conditions: Temperature and humidity affect fiber production. Low humidity can result in thicker fibers and quicker solvent evaporation, whereas high humidity can cause beads to develop and reduce the fiber diameter. Temperature affects the solvent evaporation rates and viscosity of the polymer solutions, which in turn affects the fiber shape [135].

Additives and crosslinking agents: The characteristics of the fiber can be altered by the addition of additives or crosslinking agents to the polymer solution. While crosslinking agents increase fiber stability and resistance to degradation, additives, such as surfactants or NPs, can improve mechanical qualities or introduce functions [136].

Biopolymer-based nanofibers: Biopolymers have adjustable characteristics, biocompatibility, and biodegradability, making them advantageous for therapeutic applications. Among their many applications are biosensors, scaffolds for TE, wound dressings, and DDS. Utilizing the distinct characteristics of biopolymers, including GE, collagen, alginate, and cellulose, scientists may create customized NF platforms that target certain therapeutic requirements [137].

## 9. Regulatory Consideration and Patents

The commercial and regulatory landscapes surrounding electrospinning have expanded in recent years. The relevance of electrospinning depends on the composition of the NFs. Many studies have been conducted on crosslinking treatments and the use of toxic solvents that are not FDA-approved. Operating conditions have encouraged its use, and it is a cutting-edge technology used in several industries, including the food and pharmaceutical industries. The primary issue is the scarcity of solvents that can dissolve both manufactured and natural polymers. The low volatility of certain solvents employed in the production of multiaxial fibers is a concern, and the slow production rate of 0.0010.1 g/h per spinneret in large-scale electrospinning is another issue [138]. International directives such as those established by the Food and Agriculture Organization (FAO) and the United Nations Environment Program (UNEP), which linked strategic principles with technological advancements in NF technology by incorporating the production of “green,” environmentally friendly NFs using an electrospinning method, facilitated the formulation of national and regional policies [139]. Currently, there are few formal regulatory guidance documents and unclear regulations regarding nanomedicines. A few factors that could be significant from a regulatory standpoint should be considered in the early stages of research when creating NFs as a possible medical device. The materials utilized must first meet the safety regulations. Selecting a polymer that is deemed safe and has received official approval from authorities is advised. Validation can be used to show that the electrospinning procedure and apparatus are appropriate for accurately controlled and repeatable production. Nozzle-based electrospinning is superior to nozzle-free electrospinning in this respect because the latter produces a non-uniform fiber diameter owing to simultaneous jets [139]. Polymers that are both biocompatible and biodegradable are typically preferred. Using electrospinning to innovate an existing approved product could be a wise move. To close endonasal surgical defects, Campbell et al. synthesized NFs from an FDA-approved cyanoacrylate polymer and compared them with Adherus^®^, an FDA-approved common dural sealant [140]. In general, medical devices are governed by a clearly defined regulatory framework, but the regulation of those devices that contain nanomaterials is not. It is generally accepted that medical devices that employ nanomaterials should be categorized as having a higher risk. Therefore, caution should be exercised. While the EU’s directives and applicable ISO standards are undoubtedly helpful starting points, interacting with the authorities on particular matters may also be beneficial. Following this classification, adherence to class norms is required [141]. Nonetheless, owing to its potential for use in biomedical and other nanotechnical applications, it has recently garnered considerable attention. The alteration of the electrospinning system configurations and the impact of the process parameters on the fibers, as well as how they are used for local chemotherapy, describe their use in drug administration, including carrier materials, loaded medicines, and their release kinetics. Currently, most research on the release of anti-bacterial agents and medications (such as psychotropic and antineoplastic agents) has been conducted in vitro. Comprehensive systemic investigations are required prior to considering any clinical commercialization in vivo, particularly those pertaining to the kinetics and dynamics of drug release, the impact of drug dosage and release kinetics on therapeutic efficacy, and the biodistribution of the released pharmaceuticals. Extensive research is necessary to determine the hazardous impact of polymeric carriers, as well as their distribution and removal mechanisms.

## 10. Conclusions and Future Perspective

The utilization of GE/CH-based NFs in drug delivery holds great significance for advancing biomedical applications. These biopolymers offer unique advantages including biocompatibility, biodegradability, and the ability to mimic the extracellular matrix, making them ideal candidates for tissue regeneration, wound healing, and drug delivery. Among other methods, electrospinning has proven to be effective in fabricating NFs with controlled morphology, enabling precise wound healing properties coupled with GE/CH cell adhesion and regenerative properties. However, challenges such as the optimization of fiber structures, drug-loading efficiency, and stability still remain. Despite these challenges, the potential of GE/CH-based NFs to improve drug delivery, minimize adverse effects, and enhance patient outcomes continues to drive research and innovation in this field. Furthermore, advancements in the widespread application of CH/Ge-based NFs in diverse medical fields may ultimately contribute to more effective biomedical applications. The CH/Ge-based NFs indicate the aforementioned widespread applicability due to their biocompatibility and biodegradability in nature, which ultimately makes them the most applied polymeric materials, compared to synthetic polymers.

The future of CH/Ge-based NFs in drug delivery holds great promise, particularly in enhancing targeted therapy for various complications. Advances in functionalization techniques, such as incorporating specific targeting ligands and stimuli-responsive elements, can improve the precision and control of drug release. The further optimization of drug-loading capacity and release profiles is crucial to enhance their therapeutic efficacy. Ensuring biocompatibility and safety through comprehensive studies will be vital for clinical application. Additionally, the potential for multi-drug delivery systems could offer innovative solutions for complex diseases. Scalability and cost-effective production methods will be key to making these systems commercially viable, while the combination with other nanomaterials may further enhance their therapeutic potential. Ultimately, successful clinical translation and regulatory approval will be pivotal in realizing the full potential of these nanofibers for managing a range of health complications.

## Figures and Tables

**Table 1:** Comparison of GE and chitosan as biopolymers for fabrication of nanofibers (NFs) for topical drug delivery.

Property	Gelatin	Chitosan
Source	Derived from collagen (animal-based)	Derived from chitin
Fabrication method	Electrospinning	Electrospinning
NF diameter (nm)	~100–360 nm	~50–250 nm
Surface morphology	Smooth, slightly porous	Smooth, uniform
Mechanical strength	Lower compared to CH	Moderate
Drug-loading efficiency	Moderate (better for hydrophobic drugs)	High (especially for hydrophilic drugs)
Biocompatibility	High, non-toxic for topical use	High, suitable for skin applications
Anti-bacterial properties	Limited anti-bacterial effect, but supports healing	Anti-bacterial activity (due to positive charge)

## Data Availability

Data can be made available by corresponding authors upon request.
